# High-Frequency Adventitious Shoot Regeneration from Leaf Explants of *Jatropha curcas* L.

**DOI:** 10.3390/plants15101577

**Published:** 2026-05-21

**Authors:** Bobin Liu, Jienan Chen, Lin Zhang, Meng-Zhu Lu, Jiakai Liao, Jin Zhang

**Affiliations:** 1 Jiangsu Provincial Key Laboratory of Coastal Wetland Bioresources and Environmental Protection, Jiangsu Key Laboratory for Bioresources of Saline Soils, Jiangsu Synthetic Innovation Center for Coastal Bio-Agriculture, School of Wetlands, Yancheng Teachers University, Yancheng 224051, China; liubb@yctu.edu.cn; 2 Institute of Biological and Environmental Science & Technology, Central South University of Forestry and Technology, Changsha 410004, Chinazhlin-331@163.com (L.Z.); 3 State Key Laboratory of Tree Genetics and Breeding, Research Institute of Forestry, Chinese Academy of Forestry, Beijing 100091, China; lumz@zafu.edu.cn; 4 State Key Laboratory for Development and Utilization of Forest Food Resources, Zhejiang Key Laboratory of Forest Genetics and Breeding, International Research Center for Plant Cell Wall, College of Forestry and Biotechnology, Zhejiang A&F University, Hangzhou 311300, China; 5 Basic Forestry and Proteomics Research Center, School of Future Technology, Haixia Institute of Science and Technology, Fujian Agriculture and Forestry University, Fuzhou 350002, China

**Keywords:** micropropagation, adventitious shoot regeneration, leaf explant, sodium nitroprusside, plant growth regulators

## Abstract

*Jatropha curcas* L. is an important biofuel plant, but its narrow cultivation range and low seed yield limit its large-scale commercialization. Both genetic improvement and the large-scale clonal propagation of elite genotypes require an efficient and reliable regeneration system. In this study, a high-frequency adventitious shoot regeneration protocol was developed using leaf explants from one-year-old greenhouse-grown plants derived from seeds. An L_9_(3^3^) orthogonal design was employed to optimize the concentrations of plant growth regulators (PGRs). The optimal combination for adventitious shoot induction was 1.0 mg·L^−1^ TDZ, 0.5 mg·L^−1^ IBA, and 1.5 mg·L^−1^ BA. Furthermore, the effect of sodium nitroprusside (SNP), a nitric oxide donor, was investigated. Supplementation with 2.0 mg·L^−1^ SNP significantly increased both the regeneration frequency and the shoot number per explant when compared to the control. Leaf maturity also significantly influenced the regeneration capacity, with the fourth expanded leaf at the light-green stage showing the greatest response. Under optimized conditions, including PGRs, SNP, and appropriate explant maturity, adventitious shoots were observed within 4 weeks, with a regeneration frequency of 88.0% and an average of 18.7 shoots per explant. This system provides a practical basis for the propagation and genetic improvement of *J. curcas*.

## 1. Introduction

*Jatropha curcas* L., a perennial shrub or small tree belonging to the *Euphorbiaceae* family, has garnered worldwide attention as a promising non-edible biofuel feedstock [1,2]. The plant’s seeds contain 40% to 60% oil [1,3], exceeding the oil contents of conventional oilseed crops such as rapeseed and soybean. Notably, its desirable biodiesel properties meet both American and European standards for biodiesel [1,4]. Beyond its energy applications, *J. curcas* is valued for its adaptability to marginal lands, drought tolerance, and potential for phytoremediation, underscoring its economic and ecological significance [5,6]. Additionally, its responsiveness to in vitro culture renders it a useful model for studying regeneration mechanisms in woody plants.

Despite its considerable potential, *J. curcas* remains essentially an undomesticated plant species [7,8]. As a cross-pollinated, heterozygous plant, it exhibits high phenotypic and genetic variability, resulting in significant variations in seed yield, oil content, and other agronomically important traits among seedlings [6,7,9]. This genetic heterogeneity poses a major challenge for large-scale commercial cultivation, as uniform, high-yielding planting materials are required to ensure consistent productivity and oil quality. Therefore, the development of efficient clonal propagation methods is essential for capturing and multiplying elite genotypes with desired traits, thereby preserving their genetic fidelity and ensuring a stable supply of superior planting material for biodiesel production.

In vitro plant regeneration offers a useful approach for the mass propagation of elite genotypes and serves as a prerequisite for genetic transformation studies. Among various explant types, leaf and petiole tissues have been extensively investigated for their regeneration potential in *J. curcas* [10,11,12]. Early studies demonstrated shoot organogenesis from leaf explants on Murashige and Skoog (MS) medium [13] supplemented with benzyladenine (BA) and indole-3-butyric acid (IBA), achieving regeneration frequencies of 50–80% [10,14]. Subsequent research focused on optimizing culture conditions using thidiazuron (TDZ), a potent cytokinin-like compound, which significantly improved the regeneration efficiency. Deore and Johnson (2008) reported 53.5% shoot bud induction from leaf explants on MS medium containing 0.5 mg·L^−1^ TDZ, 0.5 mg·L^−1^ BA, and 0.1 mg·L^−1^ IBA [11]. More recently, Liu et al. (2020) achieved a 78.58% regeneration frequency from leaf explants of mature plants using a 30-min treatment with 26 mg/L TDZ solution followed by culture on hormone-free medium [15]. Similarly, petiole explants have shown promising results, with regeneration frequencies reaching 65–87% under optimized conditions [12,16]. Despite these advances, the regeneration efficiency remains variable and often suboptimal, highlighting the need for further protocol refinement.

TDZ has emerged as an important PGR for *J. curcas* regeneration due to its strong cytokinin-like activity and ability to induce morphogenic responses even in recalcitrant woody species [17,18]. Numerous studies have demonstrated the effectiveness of TDZ in comparison to conventional cytokinins such as BA and kinetin in promoting adventitious shoot formation from various explants [11,12,19,20]. TDZ has been shown to modulate endogenous auxin levels, stimulate de novo synthesis of IAA and its precursor tryptophan, and increase endogenous cytokinin and ethylene contents [18]. However, the optimal concentration and exposure duration vary considerably depending on the explant type, genotype, and culture conditions. High TDZ concentrations often induce compact shoot bud clusters that fail to elongate, necessitating subsequent transfer to media with reduced TDZ or alternative PGR combinations [12,19]. The addition of sodium nitroprusside (SNP), a nitric oxide donor, promotes regeneration in other woody plants [21,22]. However, achieving optimal regeneration frequency and multiplication efficiency requires further investigation into the explant physiological status and the optimal medium. In the present study, we aimed to establish an efficient adventitious shoot regeneration system from leaf explants of *J. curcas* by optimizing PGR combinations, leaf explant maturity, and the addition of SNP. This is expected to provide a basis for the multiplication and genetic improvement of elite clones.

## 2. Results

### 2.1. Optimization of PGR Concentrations for Adventitious Shoot Induction

**Figure 1:**
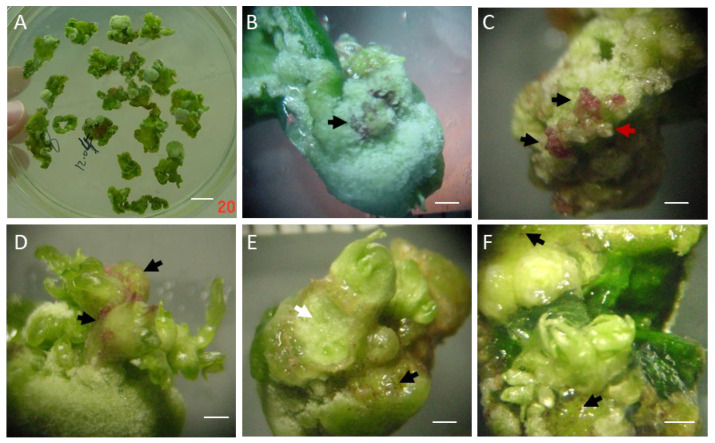
Morphology of adventitious shoot regeneration from leaf explants of *J. curcas* L. (**A**) An overview of multiple leaf explants after 5 weeks of culture, showing abundant calli at the cut edges. (**B**) Higher magnification of (**A**), showing a callus turning red in small patches (black arrows) after 5 weeks. (**C**) Adventitious shoot primordium-like structures (red arrows) emerging from these scattered red regions. (**D**) Visible shoots with reddish basal portions after 7 weeks. (**E**,**F**) Shoot emergence from a callus on the basal portion in contact with the medium, where the callus also exhibited red pigmentation (black arrows). White arrows indicate a callus at the base after adventitious shoot removal. Scale bars: 1 cm (**A**), 1 mm (**B**–**F**).

To evaluate the effects of TDZ, BA, and IBA on adventitious shoot regeneration, an L_9_(3^3^) orthogonal experiment was conducted. After 5 weeks of culture, multiple explants with abundant calli were observed at the cut edges (Figure 1A). At higher magnification, the calli were observed to turn red in small patches (Figure 1B) and, from these scattered red regions, adventitious shoot primordium-like structures emerged, particularly from the distal portion not in contact with the medium (Figure 1C). By the seventh week, visible shoots with reddish basal portions were clearly observed (Figure 1D). In addition, shoot emergence from the calli was also seen at the basal portion in contact with the medium, where the calli exhibited red pigmentation (Figure 1E,F). Thus, in this system, shoot formation was associated with callus development at the cut edges rather than arising directly from leaf tissue. The regeneration frequency for each treatment combination is presented in Table 1. Range analysis indicated that TDZ had the strongest influence (R = 38.7), followed by IBA (R = 19.4) and BA (R = 11.8), establishing the order TDZ > IBA > BA. Analysis of variance (ANOVA) further confirmed that all three factors significantly affected shoot regeneration, with TDZ exerting the most substantial effect (F = 50.23, *p* < 0.01), followed by IBA (F = 13.35, *p* < 0.01), while BA had a comparatively weaker but still significant effect (F = 4.83, *p* < 0.05) (Table 1). Among the tested combinations, the highest regeneration frequency (60.3%) was observed in treatment 5 (1 mg·L^−1^ TDZ, 0.1 mg·L^−1^ IBA, and 1 mg·L^−1^ BA). Based on marginal means, higher concentrations of each PGR generally promoted regeneration. Therefore, the theoretical optimal combination for maximizing adventitious shoot induction was 1.0 mg·L^−1^ TDZ, 0.5 mg·L^−1^ IBA, and 1.5 mg·L^−1^ BA.

### 2.2. Leaf Maturity Determines Regeneration Competence

**Figure 2:**
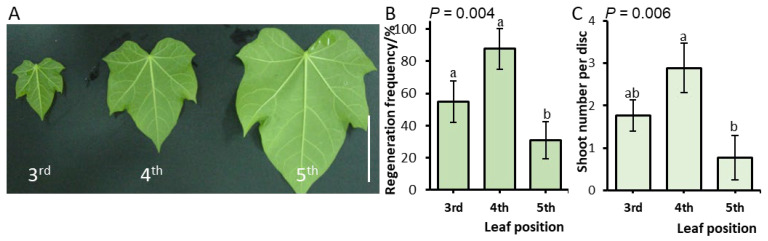
The effect of leaf age on adventitious shoot regeneration in *J. curcas* L. Cultures were maintained for 7 weeks on MS medium containing 0.5 mg·L^−1^ TDZ, 0.5 mg·L^−1^ IBA, and 1.5 mg·L^−1^ BA. (**A**) The morphology of the third to fifth leaves from the apex. Scale bar: 4 cm. (**B**) The regeneration frequency and (**C**) mean number of adventitious shoots per explant from leaf explants of different ages. Data are shown as the mean ± SD. Different letters within the same panel denote significant differences at *p* < 0.05.

To assess the effect of leaf maturity on regeneration competence in parallel with the orthogonal experiment, leaf explants from the third, fourth, and fifth fully expanded leaves (Figure 2A) were cultured under the same PGR conditions. Significant differences were observed among leaves of different maturities for both the regeneration frequency (*p* < 0.01) and the number of shoots per leaf disc (*p* < 0.01) (Figure 2B,C) after 7 weeks of culture. The fourth expanded leaf, which was light green, exhibited the highest regenerative capacity, with a regeneration frequency of 87.7% and an average of 2.9 shoots per explant (Figure 2B,C). Post hoc comparisons revealed that the fourth leaf had a significantly higher regeneration frequency than both the third and fifth leaves (Figure 2B). For shoot number, the fourth leaf produced significantly more shoots than the fifth leaf, but its advantage over the third leaf was not statistically significant (Figure 2B). These results indicate that leaf maturity influences morphogenic competence, with optimal regeneration achieved at an appropriate stage of development.

### 2.3. SNP Promotes Adventitious Shoot Regeneration at an Optimal Concentration

**Figure 3:**
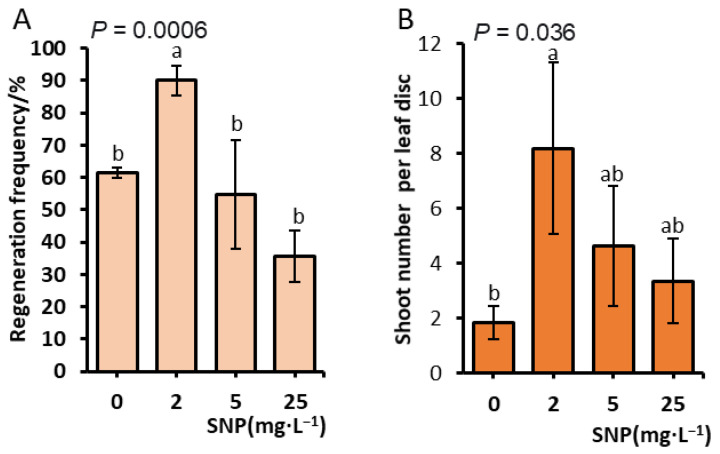
The effect of the SNP concentration on adventitious shoot regeneration in *J. curcas* L. Cultures were maintained on MS medium containing 0.5 mg·L^−1^ TDZ, 0.5 mg·L^−1^ IBA, 1.5 mg·L^−1^ BA, and SNP at 0, 2.0, 5.0, or 25.0 mg·L^−1^ for 2 weeks in darkness followed by 5 weeks under a 16 h light/8 h dark photoperiod. (**A**) Regeneration frequency. (**B**) Mean number of adventitious shoots per explant. Data are shown as the mean ± SD. Different letters within the same panel denote significant differences at *p* < 0.05.

In parallel with the orthogonal experiment (as in Section 2.2), we investigated the effect of SNP on adventitious shoot regeneration. To determine the optimal concentration of SNP for promoting adventitious shoot regeneration, leaf explants were cultured on medium supplemented with 0, 2.0, 5.0, or 25.0 mg·L^−1^ SNP. To avoid light-induced photodegradation of SNP and the associated release of toxic cyanide [23,24], explants were first incubated in the dark for 2 weeks, followed by a 5-week culture under a 16/8 h (light/dark) photoperiod (see Section 4.2). The results showed that SNP significantly influenced the regeneration frequency (*p* < 0.01), with the optimal response of 90% observed at 2 mg·L^−1^ (Figure 3A). This was significantly higher than those observed for the control and 25.0 mg·L^−1^ treatments (Figure 3A). At 25.0 mg·L^−1^ SNP, we observed that the regeneration frequency dropped markedly, and the leaf margins exhibited browning and necrosis, indicating phytotoxicity at elevated SNP levels. Subsequently, all explants were transferred to an elongation medium. As shown in Figure 3B, all SNP-treated explants produced significantly more elongated shoots per leaf disc than the untreated control (*p* = 0.036). The average number of shoots ranged from 4.7 to 8.2 in the SNP-treated groups, compared to only 1.8 in the control (Figure 3B). However, only the 2.0 mg·L^−1^ SNP treatment resulted in a statistically significant increase compared to the control (*p* < 0.05), while differences observed for the 5.0 and 25.0 mg·L^−1^ treatments did not reach significance (Figure 3B). These results indicate that SNP promotes both the regeneration frequency and the shoot multiplication capacity.

### 2.4. Effect of Optimized Combination on Adventitious Shoot Regeneration

**Figure 4:**
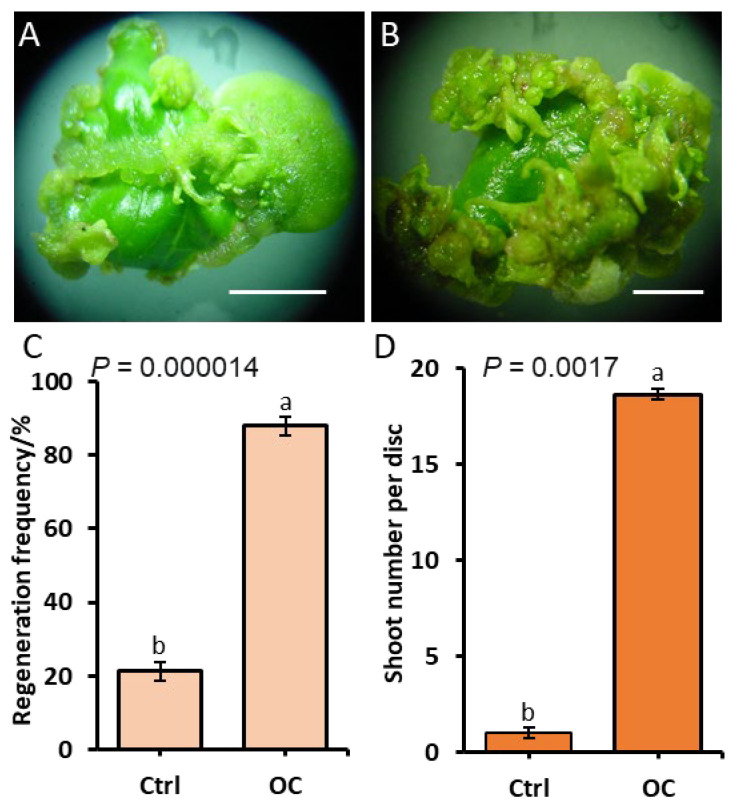
The effect of the optimized combination on adventitious shoot regeneration in *J. curcas* L. Cultures were maintained for 4 weeks (2 weeks in darkness followed by 2 weeks under a 16 h light/8 h dark photoperiod). Control treatment: MS medium with 0.5 mg·L^−1^ TDZ, 0.5 mg·L^−1^ IBA, and 1.5 mg·L^−1^ BA. Optimized treatment: MS medium with 1 mg·L^−1^ TDZ, 0.5 mg·L^−1^ IBA, 1.5 mg·L^−1^ BA, and 2 mg·L^−1^ SNP. (**A**,**B**) The morphology of adventitious shoots under control (**A**) and optimized combination (**B**) conditions. Scale bar: 5 mm. The (**C**) regeneration frequency and (**D**) mean number of adventitious shoots per explant. Data are shown as the mean ± SD. Different letters within the same panel denote significant differences at *p* < 0.05.

To test whether adding SNP to the optimal PGRs could further enhance the regeneration efficiency, we conducted a validation experiment using the fourth expanded leaf, which had been identified as the optimal explant in the leaf maturity experiment (Figure 2). Leaf explants were cultured under two conditions: a control treatment on MS medium containing 0.5 mg·L^−1^ TDZ, 0.5 mg·L^−1^ IBA, and 1.5 mg·L^−1^ BA without SNP and an optimized treatment on MS medium supplemented with 1 mg·L^−1^ TDZ, 0.5 mg·L^−1^ IBA, 1.5 mg·L^−1^ BA, and 2 mg·L^−1^ SNP. After the initial 2-week dark period followed by 2 weeks under a 16/8 h (light/dark) photoperiod, adventitious shoots in the control treatment appeared only occasionally along the cut edges of the leaf explants, with 21.3% of explants producing shoots at an average of 1.0 shoots per explant (Figure 4A,C). In contrast, the optimized combination treatment induced a significantly greater regeneration response (*p* < 0.01): shoots emerged along the entire cut edges to form dense clusters, resulting in 88.0% of explants producing an average of 18.7 shoots per explant (Figure 4B,D). These results indicate that the optimized medium containing PGRs and SNP not only achieved a high regeneration frequency of 88.0% by the fourth week but also significantly enhanced the multiplication rate in leaf explants of *J. curcas*.

### 2.5. Shoot Elongation Promotes Rooting and Survival

**Figure 5:**
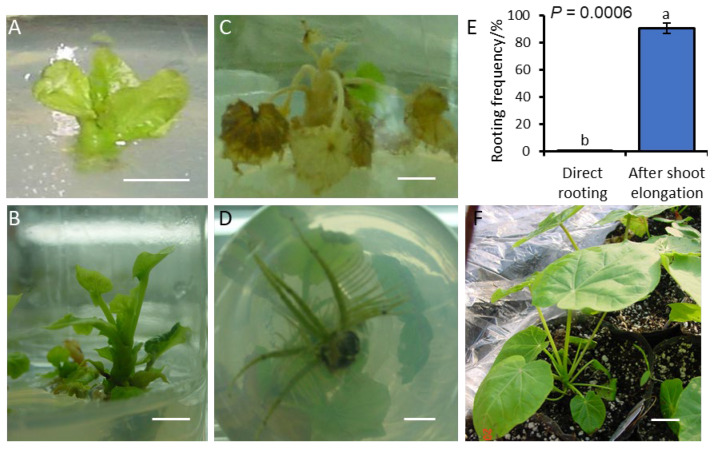
Developmental stages of adventitious shoots of *J. curcas* L. from regeneration to transplanting. (**A**) Directly regenerated shoot without elongation. (**B**) Shoot elongated on elongation medium for 2–3 weeks. (**C**) Non-elongated shoot that turned yellow and died. (**D**) Elongated shoot rooted on rooting medium. (**E**) Rooting frequency comparison between non-elongated and elongated shoots. Different letters indicate significant differences at *p* < 0.05. (**F**) Plantlet established in the greenhouse after acclimatization. Scale bar: 1 cm (**A**–**D**,**F**).

We compared adventitious shoots that were directly regenerated without elongation (Figure 5A) with those that were first elongated on elongation medium for 2–3 weeks, reaching 2–3 cm in length after 20 days of culture (Figure 5B). Shoots without elongation failed to root, turned yellow, and died (Figure 5C). In contrast, shoots that had undergone the elongation stage successfully rooted (Figure 5D), achieving a rooting frequency exceeding 90.0% (Figure 5E). The rooted plantlets were then transplanted to the greenhouse, where nearly all survived 4 weeks of acclimatization (Figure 5F).

## 3. Discussion

The optimized regeneration protocol developed in this study achieved a regeneration frequency of 88.0% with an average of 18.7 shoots per leaf disc within 4 weeks of culture (Figure 4). While this frequency is comparable to previously reported optimal frequencies for *J. curcas* leaf explants, such as 78.6% achieved by Liu et al. (2020) using a high-concentration TDZ treatment [15] and 77.6% from juvenile cotyledons reported by Khemkladngoen et al. (2011) [25], the shoot multiplication rate of 18.7 shoots per explant substantially exceeds previous reports. For comparison, Liu et al. (2020) obtained 7.3 shoots per leaf explant [15], Deore and Johnson (2008) reported 11.5 shoots per leaf disc [11], and Sujatha and Mukta (1996) achieved 10.7 shoots per leaf explant [10]. Thus, within one month of culture, our protocol achieved both a high regeneration frequency and a high shoot multiplication rate.

SNP, as a nitric oxide donor, significantly enhanced regeneration, with the optimal concentration of 2.0 mg·L^−1^ increasing the regeneration frequency to 90% (Figure 3). This aligns with previous studies demonstrating NO’s involvement in plant regeneration across various species [21,22]. Notably, the priming effect observed with SNP exposure during the induction phase represents a particularly interesting finding. Explants treated with SNP during initial culture subsequently produced 8.2 shoots per disc upon transfer to elongation medium, compared to only 1.8 shoots in untreated controls (Figure 3). This enhanced regeneration capacity suggests that nitric oxide may trigger lasting changes in cellular competence through interactions with cytokinin and auxin signaling pathways. Previous work has established that NO can modulate auxin responses and promote cell division [26], and the priming effect observed here may reflect the establishment of a more favorable hormonal balance that maintains regenerative potential during subsequent culture.

Importantly, the optimized medium containing the predicted optimal TDZ concentration (1.0 mg·L^−1^) together with 2.0 mg·L^−1^ SNP showed strong regeneration efficiency. When the fourth leaf was used for explants, the addition of 1.0 mg·L^−1^ TDZ, 0.5 mg·L^−1^ IBA, 1.5 mg·L^−1^ BA, and 2.0 mg·L^−1^ SNP produced regeneration outcomes considerably better than those observed for the control treatment (0.5 mg·L^−1^ TDZ, 0.5 mg·L^−1^ IBA, 1.5 mg·L^−1^ BA, no SNP) (Figure 4). However, it should be acknowledged that in this experimental design, the TDZ concentration and SNP addition were changed simultaneously; therefore, the individual contributions of TDZ and SNP cannot be distinguished, nor can the presence of any interactive effects be evaluated based on the current data. This is a limitation of the present study, and future research should employ factorial designs to dissect the individual and interactive effects of TDZ and SNP on adventitious shoot regeneration in *J. curcas*. While previous studies have focused on optimizing individual parameters such as PGR combinations [11,12] or explant age [10], this study’s results suggest that combining the optimal levels of each factor can achieve notable improvements. Under the optimized combination, a regeneration frequency of 88.0% was achieved within 4 weeks, which was comparable to the frequencies obtained in the orthogonal experiment after 7 weeks. This integrated approach provides practical protocols for large-scale propagation and theoretical insights into the multi-factorial control of plant regeneration.

In conclusion, this study established an efficient adventitious shoot regeneration method for *J. curcas* using leaf explants, achieving a regeneration frequency of 88.0% and 18.7 shoots per explant within 4 weeks.

## 4. Materials and Methods

### 4.1. Plant Materials

Mature seeds were collected from a local population in Fujian, China. Seedlings were grown for one year in a greenhouse at the Chinese Academy of Forestry, Beijing, and served as the source of leaf explants for all subsequent experiments. Fresh young leaves were collected, surface-sterilized using a 20% (*v*/*v*) sodium hypochlorite solution (~6.8 g L^−1^ available chlorine) (local commercial supplier)for 20 min, and subsequently rinsed three times with sterile distilled water. They were then cut into small pieces of approximately 3 mm × 3 mm as explants and placed with the abaxial side in contact with the culture medium.

### 4.2. Culture Media and General Conditions

MS medium [13] (PhytoTechnology Laboratories, Lenexa, KS, USA) with full macro- and micronutrients and vitamins, as per the original formulation, was used as the basal medium in all experiments. Each medium was supplemented with 3% (*w*/*v*) sucrose (Xilong Scientific Co., Guangdong, China) and solidified with 0.5% (*w*/*v*) plant tissue culture-grade agar (Chembase, Beijing, China). All media were adjusted to pH 5.8 to 6.2 with 1N NaOH or 1N HCl (both from Xilong Scientific Co., Guangdong, China) before autoclaving at 120 °C for 15 min. Sodium nitroprusside (SNP) (Sigma-Aldrich, St. Louis, MO, USA) was used as the nitric oxide donor. All growth regulators except SNP were added before the autoclaving. SNP was filter-sterilized (0.22 μm pore size) and added to the autoclaved medium after it had cooled to approximately 50 °C. All cultures were maintained at 23 to 25 °C under a 16 h light and 8 h dark photoperiod with a photosynthetic photon flux density of 50 μmol m^−2^ s^−1^ provided by cool white fluorescent tubes (Foshan Electrical and Lighting Co., Foshan, China). For experiments involving SNP, the cultures were kept in darkness for the first 2 weeks and then transferred to the light conditions described above. Each treatment consisted of three replicates, and each replicate contained 18–22 explants at the end of the culture period. This experimental design applied to all subsequent experiments.

### 4.3. Effect of BA, IBA, and TDZ on Adventitious Shoot Induction

To evaluate the effects of BA (Sigma-Aldrich, St. Louis, MO, USA), IBA (Sigma-Aldrich, St. Louis, MO, USA), and TDZ (Sigma-Aldrich, St. Louis, MO, USA), an L_9_(3^3^) orthogonal experiment was conducted. Explants were cultured on MS medium containing BA (0.5, 1.0, or 1.5 mg·L^−1^), IBA (0.05, 0.1, or 0.5 mg·L^−1^), and TDZ (0, 0.5, or 1.0 mg·L^−1^) under standard light conditions for 7 weeks.

### 4.4. Effect of SNP on Adventitious Shoot Induction

Explants were cultured on MS medium containing 0.5 mg·L^−1^ TDZ, 0.5 mg·L^−1^ IBA, 1.5 mg·L^−1^ BA, and SNP at 0, 2, 5, or 25 mg·L^−1^. The first 2 weeks were in darkness, followed by 5 weeks under standard light conditions (7 weeks total). After this period, all explants, regardless of whether visible shoot clusters had formed, were transferred to elongation medium (MS medium with 1.5 mg·L^−1^ BA, 0.05 mg·L^−1^ IBA, and 0.05 mg·L^−1^ GA_3_ (Sigma-Aldrich, St. Louis, MO, USA)) [27]. The number of elongated shoots (>1 cm) per original explant was recorded after 20 days.

### 4.5. Effect of Leaf Maturity on Adventitious Shoot Induction

Leaf maturity was defined by leaf position from the apex for the third leaf (pale green, not fully expanded), the fourth leaf (light green, fully expanded), and the fifth leaf (dark green, fully expanded) (Figure 3A). Explants from each position were cultured on MS medium containing 0.5 mg·L^−1^ TDZ, 0.5 mg·L^−1^ IBA, and 1.5 mg·L^−1^ BA under standard light conditions for 7 weeks.

### 4.6. Optimized Combination Treatment

The orthogonal, SNP, and leaf maturity experiments were conducted in parallel; thus, the predicted optimal PGR combination was not yet known during the individual screenings. For the validation experiment, the fourth leaf was used for explants. The control treatment used MS medium with 0.5 mg·L^−1^ TDZ, 0.5 mg·L^−1^ IBA, and 1.5 mg·L^−1^ BA (no SNP). The optimized treatment used MS medium with 1 mg·L^−1^ TDZ, 0.5 mg·L^−1^ IBA, 1.5 mg·L^−1^ BA, and 2.0 mg·L^−1^ SNP. Explants were kept in darkness for 2 weeks, followed by 2 weeks under standard light conditions (4 weeks total).

### 4.7. Shoot Elongation, Root Induction, and Transplantation

For elongation, adventitious shoots were transferred to MS medium with 0.5 mg·L^−1^ BA and 0.01 mg·L^−1^ IBA for 20 days. Elongated shoots (2–3 cm) were then transferred to rooting medium (MS medium with 0.3 mg·L^−1^ IBA) for 30 days. Rooted plantlets were transplanted to pots containing peat /perlite (both sourced from local suppliers in China) (3:1, *v*/*v*) and acclimatized in a greenhouse under 70–80% relative humidity.

### 4.8. Statistical Analysis

The regeneration frequency (%) was calculated as (explant number with shoots/total explant number) × 100. Normality and homogeneity of variance were tested prior to analysis. Regeneration frequency data were arcsine square-root-transformed before ANOVA; the results were consistent with those of non-parametric Kruskal–Wallis tests. Significant differences among means were determined by means of ANOVA followed by Tukey’s HSD multiple range test at *p* < 0.05. For comparisons between two groups, the independent samples *t*-test (two-tailed) was applied. Equality of variances was assessed using Levene’s test; when variances were unequal, Welch’s correction was employed. Data are expressed as the mean ± SD. SPSS 16.0 (IBM Corp., Armonk, New York, NY, USA) was used.

## Figures and Tables

**Table 1:** Effect of BA, IBA, and TDZ on adventitious shoot regeneration in *J. curcas* L.

Code	BA /(mg·L^−1^)	IBA /(mg·L^−1^)	TDZ /(mg·L^−1^)	Regeneration Frequency/%	Factor	Range Analysis (R)		Factor ANOVA (F/P)
1	0.5	0.05	0	1.7 ± 2.9 d	BA	R = 11.8 27.7 ± 2.7 b		4.83 *p* < 0.05
2	0.5	0.1	0.5	32.8 ± 8.0 bc	IBA	R = 19.4		13.35 *p* < 0.01
3	0.5	0.5	1	48.5 ± 6.1 ab	TDZ	R = 38.7 39.5 ± 2.7 a		50.23 *p* < 0.01
4	1	0.05	0.5	20.6 ± 8.5 cd				
5	1	0.1	1	60.3 ± 4.2 a				
6	1	0.5	0	20.0 ± 9.5 cd				
7	1.5	0.05	1	44.6 ± 4.0 ab				
8	1.5	0.1	0	17.2 ± 8.9 cd				
9	1.5	0.5	0.5	56.6 ± 4.0 a				

Note: Different lowercase letters within the same column denote significant differences at *p* < 0.05.

## Data Availability

The original contributions presented in this study are included in the article. Further inquiries can be directed to the corresponding authors.

## Abbreviations

BA	6-Benzylaminopurine
IBA	Indole-3-butyric acid
MS	Murashige and Skoog medium
TDZ	Thidiazuron
NO	Nitric oxide
SNP	Sodium nitroprusside
ANOVA	Analysis of variance
PGR	Plant growth regulator
